# Supplementary figures and images for: E-cigarettes versus nicotine patches for perioperative smoking cessation: a pilot randomized trial
Source: PeerJ. 2018 Sep 28;6:e5609. doi: 10.7717/peerj.5609 (PMC6166615; doi:10.7717/peerj.5609)

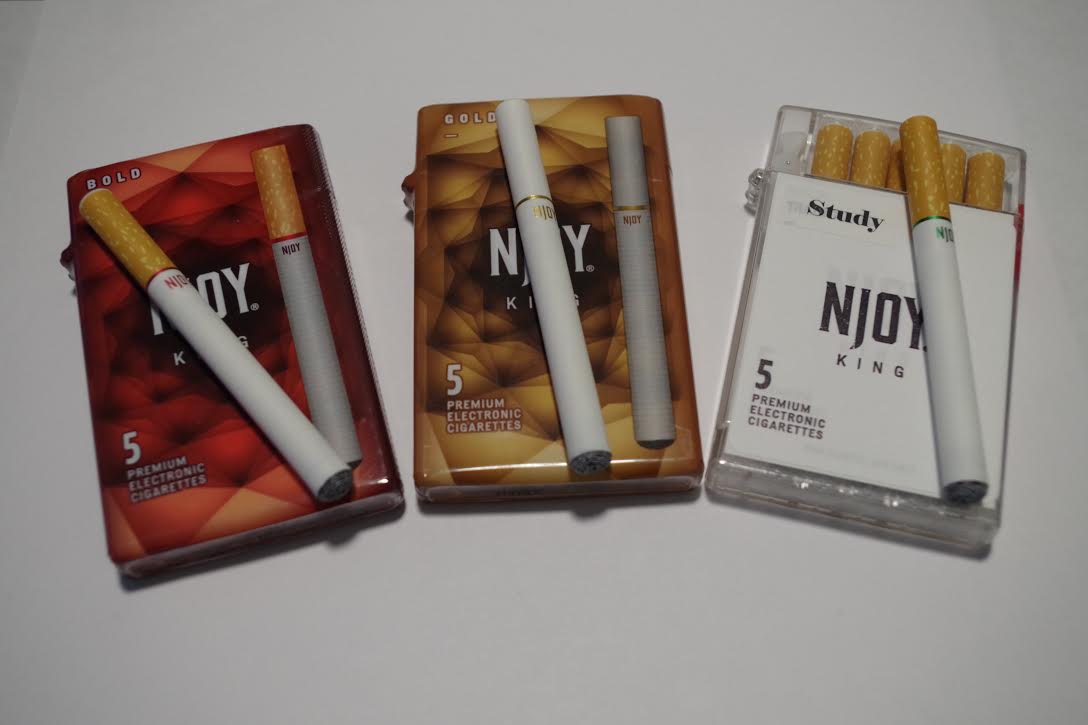

Supplement: Figure S1 — Each participant in the END group was issued 6-weeks of NJOY e-cigarettes, pictured above. The number of e-cigarettes issued corresponded to their baseline smoking, with 1 NJOY e-cigarette equivalent to 10 cigarettes per day. Participants were given 3 weeks of Bold (4.5%), 2 weeks of Gold (2.4%) and 1 week of Study (0%) e-cigarettes and instructed to use them ad libitum in lieu of their usual cigarettes. END=electronic nicotine delivery. This photograph was taken by the author Susan M. Lee. [file peerj-06-5609-s001.jpg]

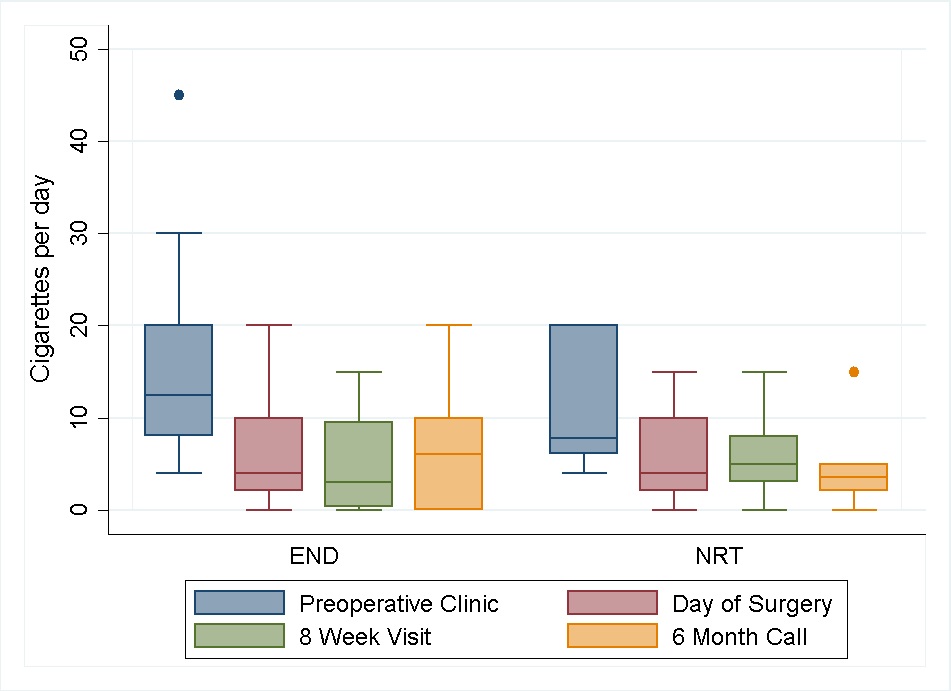

Supplement: Figure S2 — Boxplots indicating distribution of cigarettes smoked per day at baseline (Preoperative Clinic visit), day of surgery, 8-week, and 6-month follow-up visits. There were no statistically significant differences between groups END and NRT at any time points. However, both END and NRT groups had significantly lower cigarette consumption at each follow-up visit compared to baseline, as compared using Wilcoxon Signed-Rank tests (p = 0.0004, 0.0001, and 0.0004 for END group comparing baseline visit to day of surgery, 8-week and 6-month follow-up visits respectively; p = 0.02, 0.01, and 0.008 for NRT group comparing baseline visit to day of surgery, 8-week and 6-month follow-up visits respectively). END, electronic nicotine delivery; NRT, nicotine replacement therapy. [file peerj-06-5609-s002.jpg]
