# Supplementary material for: E-cigarettes versus nicotine patches for perioperative smoking cessation: a pilot randomized trial
Source: PeerJ. 2018 Sep 28;6:e5609. doi: 10.7717/peerj.5609 (PMC6166615; doi:10.7717/peerj.5609)
Supplement: Supplemental Information 1 [file peerj-06-5609-s003.docx]

Contents

[1. Grant Mechanism 1](#_Toc403569595)

[2. P.I. Name 1](#_Toc403569596)

[3. Grant Administrator 2](#_Toc403569597)

[4. Research Mentors / Co-investigators 2](#_Toc403569598)

[5. Project title 2](#_Toc403569599)

[6. Abstract 2](#_Toc403569600)

[7. Proposal 2](#_Toc403569601)

[SPECIFIC AIMS: 3](#_Toc403569602)

[Background and Significance: 3](#_Toc403569603)

[Preliminary studies: 4](#_Toc403569604)

[Detailed Experimental Design and Methods: 4](#_Toc403569605)

[Control and intervention groups: 5](#_Toc403569606)

[Outcomes: 6](#_Toc403569607)

[Qualitative Method: 6](#_Toc403569608)

[Sample size estimation: 6](#_Toc403569609)

[Randomization and blinding: 6](#_Toc403569610)

[Techniques to maximize adherence: 7](#_Toc403569611)

[Adverse events, side effects, and interim monitoring: 7](#_Toc403569612)

[Ethical considerations: 7](#_Toc403569613)

[Statistical analysis plan: 7](#_Toc403569614)

[Qualitative Analysis: 8](#_Toc403569615)

[Timetable: 8](#_Toc403569616)

[How this pilot study will lead to future funding: 8](#_Toc403569617)

[Mentoring plan: 8](#_Toc403569618)

[Strengths of this research plan: 9](#_Toc403569619)

[Literature cited: 10](#_Toc403569620)

[16. Budget 12](#_Toc403569621)

# 1. Grant Mechanism

UCSF Department of Anesthesia and Perioperative Care Internal Funds - $18,569

UCSF Resource Allocation Program (RAP) - $29,969

# 2. P.I. Name

Susan Ming Lee, MD, FRCPC

Clinical Instructor

UCSF Department of Anesthesia and Perioperative Care

email address: [lees4@anesthesia.ucsf.edu](mailto:lees4@anesthesia.ucsf.edu)

phone: 415-684-3221

# 3. Grant Administrator

Clair Harmon

Grants & Contracts Analyst

UCSF Department of Anesthesia and Perioperative Care

email address: [harmonc@anesthesia.ucsf.edu](mailto:harmonc@anesthesia.ucsf.edu)

phone: 415-476-4762

# 4. Research Mentors / Co-investigators

1) **Art Wallace, MD, PhD**

Professor, UCSF Department of Anesthesia and Perioperative Care

Chief, Anesthesiology Service

San Francisco Veterans Administration Medical Center (SFVAMC)

email address: [art.wallace@va.gov](mailto:art.wallace@va.gov)

phone: 415-750-2069

2) **Mehrdad Arjomandi, MD**

Assistant Professor in Residence, UCSF Department of Medicine

Division of Pulmonary, Critical Care, Allergy, Immunology, and Sleep Medicine

email: [mehrdad.arjomandi@ucsf.edu](mailto:mehrdad.arjomandi@ucsf.edu)

phone: 415-221-4810 ext. 4393

5. Project title: A Pilot Randomized Controlled Clinical Trial - "**E**lectronic **N**icotine **D**elivery device (e-cigarette) for **perioperative** **smoking** cessation in veterans" (The END Perioperative Smoking Pilot Study)

6. Abstract

Cigarette smoking is known to increase the risk of complications, and even mortality, in patients undergoing surgery. Despite this knowledge, and the fact that surgery is a 'teachable moment' that may motivate patients to quit, it is unclear what clinicians can do to minimize this risk. Not only do veterans smoke more than the general population, but they may also face unique challenges in quitting. E-cigarettes have been proposed as an alternative to nicotine replacement therapy as an aid to smoking cessation that may be more acceptable to some patients. However, there is currently a desperate need for more efficacy and safety data on the use of e-cigarettes.

The purpose of this pilot randomized trial is to determine the feasibility of e-cigarettes and telephone counselling (compared to transdermal nicotine replacement and telephone counselling) as a harm-reduction tool that may lead to increased smoking cessation in the perioperative setting in smokers presenting for elective surgery at the San Francisco Veterans Affairs Medical Center. Secondary outcomes include acceptability of e-cigarettes over transdermal nicotine replacement, length-of-stay in the post-anesthesia care unit, hospital length-of-stay, postoperative complications within the first 30-days, and smoking status 8-weeks after randomization. This pilot study is designed to provide the preliminary data necessary to plan and fund a larger-scale randomized clinical trial that will assess the utility of e-cigarettes in achieving smoking cessation perioperatively. Our ultimate goal is to add to the limited existing data on the safety and efficacy of e-cigarette use in smoking cessation, specifically in the perioperative setting where the risks of continued smoking are great and the motivation to stop is high.

7. Proposal

**Title:** A Pilot Randomized Controlled Clinical Trial - "**E**lectronic **N**icotine **D**elivery device (e-cigarette) for **perioperative** **smoking** cessation in veterans" (The END Perioperative Smoking Pilot Study)

## SPECIFIC AIMS:

It is well-known that smokers suffer more complications and higher risk of mortality after surgery than non-smokers[^1^](#_ENREF_1)^,^[^2^](#_ENREF_2). Despite this knowledge, it is unclear what clinicians can do to minimize this risk. Surgery represents a 'teachable moment' that might encourage smokers to engage in permanent cessation[^3^](#_ENREF_3). Several small trials have shown that smoking cessation interventions can increase smoking cessation and reduce postoperative complications, particularly wound-healing complications, which can have an absolute risk reduction of up to 25%[^4-7^](#_ENREF_4). Smoking cessation initiated in the perioperative period can also promote long-term smoking cessation[^7-9^](#_ENREF_7). Despite the benefits of comprehensive smoking cessation interventions including nicotine replacement therapy, current standard of care at the SFVAMC does not routinely include specific preoperative smoking cessation pharmacotherapy or counselling. Although there is an urgent need for more data, e-cigarettes have been proposed as an alternative to nicotine replacement therapy that are at least as effective for smoking cessation[^10^](#_ENREF_10), and may be more acceptable to some patients[^11^](#_ENREF_11).

The main hypothesis of this pilot study is that the use of e-cigarettes and telephone counselling, compared to telephone counselling and transdermal nicotine replacement, in the perioperative period results in increased smoking cessation on the day of surgery and at 8-weeks after randomization in smokers presenting for elective surgery. As secondary hypotheses, the study will also assess the acceptability of e-cigarettes versus nicotine patches, postoperative complications within the first 30-days, length-of-stay in the PACU and hospital length-of-stay. We will examine the above hypotheses through the following aims:

**» Aim 1) To determine how e-cigarettes plus counselling compare to transdermal nicotine replacement plus counselling for the achievement of smoking cessation, when introduced prior to elective surgery in veterans.**

We plan to carry-out a pilot randomized controlled trial with parallel design comparing e-cigarettes and telephone counselling with transdermal nicotine replacement and telephone counselling. Our primary outcome is smoking cessation on the day of surgery, as confirmed biochemically by exhaled carbon monoxide. Smoking reduction (self-reported cigarettes per day) of 50% or more and bedside spirometry readings will be assessed as secondary outcomes.

**» Aim 2) To determine the acceptability of e-cigarettes amongst veterans as an aid for smoking cessation and to determine the feasibility of recruitment, randomization, and follow-up procedures in preparation for large-scale trial.**

Through implementation of this pilot trial, we will determine the feasibility and acceptability of e-cigarettes for smoking cessation perioperatively in the veteran population and obtain the preliminary data necessary to run a larger trial on the effectiveness of e-cigarettes as a perioperative smoking cessation aid.

**» Aim 3) To determine the safety of e-cigarettes as a harm reduction strategy to achieve short-term perioperative smoking cessation.**

We plan to improve the overall knowledge of the safety of short-term e-cigarettes use through careful surveillance for adverse events and side effects.

**» Aim 4) To determine if e-cigarette use preoperatively is associated with a lower risk of complications postoperatively.**

We plan to measure the following secondary outcomes: postoperative complications and mortality within the first 30 days, post-anesthesia care unit (PACU) length-of-stay, and hospital length-of-stay. This will help us understand if e-cigarettes have the potential to be used for harm-reduction perioperatively.

## Background and Significance:

Importance of the problem: Cigarette smoking is known to increase the risk of complications in patients undergoing surgery[^12-15^](#_ENREF_12). Cigarette smoking is more prevalent in United States veterans than non-veterans, being reported by 25% of veterans surveyed in 2007, compared to 20% of non-veteran adults[^16^](#_ENREF_16). Veterans may face unique challenges in smoking cessation, given the perception that smoking was a normalized part of military life[^17^](#_ENREF_17) and the high rate of coexisting mental health disorders[^18^](#_ENREF_18). An analysis within the Veterans Affairs (VA) healthcare system showed that pulmonary complications, cardiovascular complications, and surgical site infections were mediators of smoking-associated mortality at 6-months and 1-year after elective surgery[^19^](#_ENREF_19). A systematic review has shown that preoperative smoking cessation therapy improves both short and long-term smoking cessation[^20^](#_ENREF_20). In fact, a surgical encounter with the healthcare system has been described as a “teachable moment” that may provide extra motivation for patients to permanently stop smoking. Despite this information, most anesthesiologists do not routinely offer smoking cessation advice to their patients[^21^](#_ENREF_21)^,^[^22^](#_ENREF_22).

Innovative nature of the proposed research: There is currently a desperate need for more data regarding the use of e-cigarettes and their role in smoking cessation interventions[^23^](#_ENREF_23). Despite the fact that the FDA has not approved any e-cigarettes for therapeutic use, e-cigarettes are widely marketed with cessation-related claims[^24^](#_ENREF_24) and have the potential to bridge the gap between smoking cigarettes and abstaining. The limited evidence we do have is that e-cigarettes are modestly effective, similar to transdermal nicotine replacement, in achieving smoking cessation at 6-months with few significant adverse events[^10^](#_ENREF_10). A review of e-cigarettes showed that only 4 clinical trials have examined the efficacy of e-cigarettes for smoking cessation, 3 of which did not have a control group[^24^](#_ENREF_24). Despite this paucity of data, a survey of 112 preoperative patients showed that 55% had tried e-cigarettes and 71% of those that had tried stated that their reason was to quit smoking[^25^](#_ENREF_25). The current study will attempt to determine the acceptability of e-cigarettes as a smoking cessation aid and add to the limited existing data on the safety and efficacy of e-cigarette use in smoking cessation, specifically in the perioperative setting where the risks of continued smoking are great and the motivation to stop is high.

Next steps and potential impact of findings: The findings from this study will demonstrate the feasibility and acceptability of e-cigarettes as a smoking cessation aid perioperatively, providing preliminary data that will allow for larger studies and improved understanding of the safety and efficacy of e-cigarette use, not only in the perioperative environment, but also more broadly in a public health context. Furthermore, current literature indicates that dual use of e-cigarettes and conventional cigarettes is common[^24^](#_ENREF_24) and may reduce the potential harm-reduction role e-cigarettes might play in the broader public health context, since light and intermittent smoking of conventional cigarettes still conveys a significant mortality risk compared to abstinence[^26^](#_ENREF_26). Previous studies of e-cigarette use for smoking cessation have not specifically counselled patients to refrain from dual use, so this study will contribute by determining how commonly dual use occurs in a controlled setting.

## Preliminary studies:

**Research Environment and Feasibility**: SFVAMC provides a unique environment to study smoking cessation initiated in a perioperative setting. Considering only surgical admissions at the SFVAMC in 2011, 21.9% had an International Classification of Diseases 9 (ICD-9) code of 305.1, indicating tobacco use during the year of surgery, which demonstrates the burden of tobacco addiction among veterans and supports the feasibility of our proposed research plan. These rates also highlight the relevance and importance of studying smoking cessation in the VA population. Furthermore, the surgical encounter is a time when veterans are engaged in healthcare and represents an opportune moment for a dedicated smoking cessation intervention. SFVAMC cares for almost 4000 surgical patients per year, making it an ideal site to study perioperative smoking cessation interventions.

**Ability to Implement Clinical Trials:** Our research team has extensive experience with clinical trials initiated during perioperative care[^4^](#_ENREF_4)^,^[^27-36^](#_ENREF_27). In particular, Dr. Lee has developed and implemented a randomized trial of a perioperative smoking cessation intervention[^4^](#_ENREF_4). Dr. Wallace has extensive expertise in the development and implementation of clinical trial research protocols to reduce perioperative risk ([**www.betablockerprotocol.com**](http://www.betablockerprotocol.com)). Dr. Arjomandi is a member of the UCSF Center for Tobacco Control Research and Education with interests in the health effects of environmental toxins, including smoking. These prior projects demonstrate the ability of our research team to develop protocols, test them in randomized controlled trials, implement them in hospital systems, and demonstrate safety and efficacy in routine clinical use.

## Detailed Experimental Design and Methods:

**Study Design:** Randomized controlled trial with 2 parallel arms:

1) Electronic nicotine delivery device group (ENDD) + referral to the California Smokers' Helpline

2) Control group (referral to the California Smokers' Helpline plus transdermal nicotine replacement)

**Study population:** Adults presenting for elective surgery at the SFVAMC.

**Identification of subjects:** Potential subjects will be identified on the basis of operating room bookings and current smoking history at their preadmission evaluation in the Anesthesia Preoperative Clinic (APO clinic). We will aim to recruit patients at least 1 week preoperatively, but accept patients up to 3 days preoperatively so as to maximize the number of available subjects, but allow enough time to begin smoking cessation preoperatively. They will be seen for further evaluation of inclusion and exclusion criteria at the time of their usual preoperative assessment in the APO clinic, which according to a recent quality improvement audit, was a median of 6 days preoperatively.

**Inclusion criteria:** adults (age >18), any gender, scheduled to undergo elective surgery at the SFVAMC. Daily smokers based on self-report of at least 2 cigarettes/day and having smoked in the last 7 days. Presenting to APO clinic at least 3 days preoperatively.

**Exclusion criteria:**

Emergency surgery (booked <24 hours preoperatively)

Consumers of non-cigarette forms of tobacco only (pipe, smokeless tobacco) or marijuana only

Already enrolled in a smoking cessation trial or current smoking cessation pharmacotherapy

Already daily users of e-cigarettes or previous adverse reaction to e-cigarette or transdermal nicotine

Poor proficiency of English language**¸**as indicated by need for an interpreter (including family members) at the preadmission visit

Lacking capacity for consent (e.g. due to mental illness or dementia)**,** as indicated by consent for surgery and other medical procedures being obtained from a substitute decision maker

Pregnant or breastfeeding

Unstable cardiac condition (unstable angina, unstable arrhythmia)

## Control and intervention groups:

**1) Electronic nicotine delivery device group (ENDD) + referral to the California Smokers' Helpline**

The ENDD group will receive:

i) 6-week supply of disposable "NJOY" ENDDs (e-cigarettes)

-the number of e-cigarettes will be determined by equating the number of e-cigarettes to the number of cigarettes smoked per day (1 pack per day = 2 e-cigarettes per day = 14 e-cigarettes/week)

-veterans will be given detailed instructions for use and also instructed to start with the high nicotine content (4.5%) strength for three weeks, then decrease to the low nicotine content (2.4%) for two weeks, then switch to nicotine-free for the final week

ii) referral to the California Smokers' Helpline

-the California Smokers' Helpline is a telephone helpline offering smoking cessation counselling 7am-9pm weekdays and 9am-5pm weekends. Counselling includes individualized counselling and quit plan development, strategies to prevent relapse, and follow-up calls.

iii) brief advice from the research assistant lasting less than 2 minutes, typically a few sentences such as the following:

“The most important advice I can give you is that quitting smoking is the number one thing you can do for your health. Quitting smoking before surgery may reduce complications around surgery and there’s evidence that the longer you quit before your surgery, the less complications you’ll have. That's why your surgeon and anesthesiologist have recommended that you stop smoking as soon as possible. I encourage you to make use of the resources that have been provided to you to help you quit and to set your quit date for as soon as possible.”

iv) a brochure from the American Society of Anesthesiologists (ASA) about quitting smoking before surgery (available free from the ASA).

**2) Control group (referral to the California Smokers' Helpline + transdermal nicotine replacement therapy)**

The control group will receive the same counselling as the intervention group, but will receive a prescription for transdermal NRT instead of ENDD:

A prescription for 6 weeks of transdermal nicotine replacement (on-formulary at the VA) in the following doses: For smokers of 10 cigarettes per day or more, a 3-week supply of 21 mg/d, 1-week supply of 14 mg/d, 1-week supply of 7 mg/d, and 1-week of 0mg/d. Smokers of <10 cigarettes per day, 3 weeks of 14 mg/d patches 2 weeks of 7 mg/d patches, and 1-week of 0mg/d.

As with the intervention group, the control group will receive:

i) referral to the California Smokers' Helpline, ii) brief advice lasting less than 2 minutes, iii) a brochure from the ASA about quitting smoking before surgery

Table 1. Summary of study intervention

| **Study intervention** | **Intervention group (ENDD)** | **Control group** |
| --- | --- | --- |
| E-cigarettes (6-weeks) | YES | × |
| Transdermal NRT (6-weeks) | × | YES |
| Referral to the California Smokers' Helpline telephone helpline | YES | YES |
| Brief advice | YES | YES |
| ASA brochure | YES | YES |

## Outcomes:

**Primary outcome:** Smoking status on the day of surgery (48-hour point-prevalence abstinence), by self-report and confirmed with exhaled carbon monoxide (CO), which normalizes (to <10 ppm) within 24 hours after smoking cessation and should not be elevated with e-cigarette use only.

**Secondary outcomes:**

1. Descriptive outcomes of proportion of veterans eligible for inclusion, reasons for exclusion, and proportion of eligible veterans consenting to participate. Acceptability outcomes of satisfaction and use of intervention (any versus continuous) 8-weeks after randomization.

2. Smoking status at 8-weeks after randomization, as confirmed by exhaled CO.

3. Smoking reduction on the day of surgery and at 8-weeks after randomization (defined as 50% or less of baseline cigarettes smoked per day). Dual use of e-cigarettes and conventional cigarettes will also be assessed.

4. Bedside spirometry FEV1 (forced expiratory volume in first second), FVC (forced vital capacity), and cotinine level at 8-weeks after randomization (compared to baseline).

5. Postoperative complications or mortality within 30-days (by telephone self-report + chart review)

6. PACU length-of-stay

7. Hospital length-of-stay

8. Long-term assessment at 6 months - self-reported smoking status, including dual use of e-cigarettes.

## Qualitative Method:

A semi-structured interview guide will be used at the 8-week post-randomization visit. Interviews will be audio-recorded and transcribed verbatim for subsequent analysis. The following key questions will be asked during the interview:

--> how important were e-ciagrettes/patch to you in helping you quit (what aspects)?

--> if attempting to quit again, what technique would you try and why?

--> how important was telephone counselling to you in helping you quit (what aspects)?

--> what was the most difficult challenge in quitting (what helped you overcome it)?

--> Do you smoke non-tobacco products (e.g. marijuana)? How does this effect your quitting smoking?

If not quit:

--> how interested are you in quitting now? (why this may or may not be a good time to quit)

--> how interested would you be in trying to quit another time? (why this time is preferred)

What benefits do you think you got/will get from quitting around the time of surgery?

What did you do when you had a craving to smoke a cigarette?

For e-cigarette group:

Did you use e-cigarettes and conventional cigarettes on the same day?

What made you want to use e-cigarettes versus conventional cigarettes? (what do you smoke now?)

How was this quit attempt different/similar to previous quit attempts?

Did you use non-study e-cigarette products?

## Sample size estimation:

Previous perioperative studies have shown an absolute difference in smoking cessation rates between intervention and control groups to be 13-76%, with quit rates in the control groups ranging from 4-65%[^4-7^](#_ENREF_4). The proposed sample size is 30 (20 intervention, 10 control), balancing cost against precision, as is conventional for a pilot study. We realize that since the sample size is small, between-group differences may not be statistically significant. However, the proposed sample size will provide rough estimates of Aim 1 (smoking cessation) and Aim 4 (postoperative complications) treatment effects, residuals, and within-subject correlations needed for the full-scale trial. The sample size will also establish the acceptability of the intervention (Aim 2). In order to proceed with a full trial assigning ENDD use to a larger number of veterans, we would like to determine from this pilot study if ENDD is more acceptable to patients than NRT, as reported in previous qualitative studies[^11^](#_ENREF_11). The proposed sample size will have 80% power to detect an effect size of 1.085, accepting an alpha of 0.05 and assuming the standard deviation of a likert scale (0-7) of satisfaction with the intervention (ENDD or NRT) is around 1.0[^37^](#_ENREF_37). Even if we are underpowered to see smaller differences, the point estimates and confidence intervals will provide rough estimates that will be useful in planning a future study.

## Randomization and blinding:

After collection of baseline data, randomization will be done by computer-generated scheme with randomly permuted block sizes of 3 or 6 in a 2:1 fashion. Allocation will be concealed by consecutively numbered, sealed, opaque envelopes. Due to the nature of the intervention, blinding of subjects is not possible. whenever possible, healthcare providers will be blinded throughout the perioperative period. However, patients may report being in the study to their providers, which may inadvertently cause unblinding. Outcome adjudicators will be blinded.

## Techniques to maximize adherence:

To maximize follow-up, patients will be provided with an incentive of $40 to participate in the study to its completion. To ensure research staff adhere to the protocol, the principal investigator (PI) or co-investigator (CI) will personally observe the recruitment, consent, randomization, and intervention for the first 3 patients enrolled. The duration, frequency, and timing of all in-person sessions and level of subject’s engagement (number and type of questions) during the session will be recorded by the research staff.

## Safety of NJOY e-cigarettes:

E-cigarettes in the USA are not FDA-regulated as drugs or medical devices and there is limited safety data regarding e-cigarettes. However, a recent systematic review of worldwide e-cigarettes determined that e-cigarettes are a less harmful alternative to smoking and significant health benefits are expected in smokers who switch from tobacco to electronic cigarettes[^38^](#_ENREF_38). Specifically, there were no serious adverse events reported in the two randomized controlled trials that have included detailed e-cigarette safety analyses. There are published data on NJOY e-cigarettes[^39^](#_ENREF_39), including a study that showed that NJOY King Bold disposable e-cigarettes (same type proposed for use in this study) when used in the short-term did not have any serious adverse events in a sample of 29 regular smokers that did not intend to quit. All but one of the 15 adverse events were mild and local, with most resolving within the first few days of use as subjects became more familiar with the product. The most common adverse events were mouth, throat, or airway irritation (7), followed by cough (2) and dry throat (1). One subject experienced intermittent headaches and one subject experienced a burning sensation on lips. The only "moderate" adverse effect was throat irritation that required discontinuing use of the product. Other findings in the study included reduced exhaled carbon monoxide levels, cravings for cigarettes and withdrawal symptoms. Mild heart-rate elevation of up to 5 beats per minute in the first 10 minutes of use was noted. After one-week of use, mean cigarette use was reduced in 89% of subjects.

NJOY e-cigarettes are easily and widely available, both online and in shops that sell cigarette products, from small local stores to large chains like Costco or Wal-mart. Despite the fact that there are no e-cigarettes in the United States that are FDA approved for smoking cessation, a content analysis of e-cigarette retail website marketing found that 95% of websites made explicit or implicit health-related claims, 64% had a smoking cessation-related claim and 22% featured doctors[^40^](#_ENREF_40). Preoperative patients believe that e-cigarettes can help them quit smoking, as shown in a survey of 112 elective surgery patients, where 71% of respondents cited smoking cessation as a the reason they had tried e-cigarettes and 62% of respondents felt switching to e-cigarettes would improve their health around the time of surgery[^25^](#_ENREF_25).

## Adverse events, side effects, and interim monitoring:

Subjects will be asked both open-ended and checklist-based questions to monitor for safety.

Safety data regarding e-cigarette use is limited, but the following have been reported and will be monitored:

-headache, nausea, oropharynx irritation, dry cough (persistent or intermittent)

-pneumonia (requiring antibiotics, requiring hospitalization)

Patients will be able to contact a member of the research team by pager at any time to report adverse events and seek assistance. We do not plan unmasked interim monitoring. However, if any catastrophic adverse event is suspected to be related to the study (deemed to be life-threatening), we will unblind that subject for safety and the study will be stopped early if the life-threatening adverse event is thought to be directly related to the study intervention. Dr. Brian Cason, SFVAMC anesthesiologist with extensive experience in clinical research and research ethics, has agreed to serve as an independent data safety monitoring officer for the study.

## Ethical considerations:

Ethics approval from the UCSF committee on human research institutional review board and SFVAMC will be obtained prior to commencement of the study. Written, informed consent, will be obtained by all patients prior to trial enrollment. E-cigarettes are a widely available commercial product that are often marketed and used as a means for current smokers to quit smoking[^41^](#_ENREF_41). Recently, the FDA has issued a proposed rule that would extend the agency’s tobacco authority to cover additional products that meet the legal definition of a tobacco product, such as e-cigarettes; however, currently, e-cigarettes are not an FDA-approved form of nicotine replacement therapy (<http://www.fda.gov/newsevents/publichealthfocus/ucm172906.htm>; accessed 9/22/2014). The use of e-cigarettes may be associated with harms greater than those of NRT. These risks will be told verbally and in written format to potential study participants. The largest randomized trial to date (657 participants) investigating e-cigarettes versus nicotine patches did not find an increased adverse event rate in the e-cigarette group, indicating that short-term use of e-cigarettes is likely safe, although the adverse event rate may differ in the veteran population[^10^](#_ENREF_10). Furthermore, there is variability in the quality of commercially available products and the most-studied brand of e-cigarette is not widely available in the USA. We have therefore chosen to study a brand that has at least some published experimental and safety data.

Currently, smokers presenting for surgery receive brief pre-operative advice, but no other intervention regarding smoking cessation. Thus, even if e-cigarettes are not more beneficial than NRT, both arms of the study are receiving care that is likely better than the current standard, due to the incorporation of counselling in both the control and intervention groups.

## Statistical analysis plan:

Descriptive statistics to assess feasibility:

-proportion ineligible for inclusion (95% CI by bionmial exact) and reasons for exclusion

-proportion of eligible veterans declining to participate (95% CI by binomial exact)

Baseline characteristics to be collected prior to randomization: age, gender, race/ethnicity, comorbidities (hypertension, coronary artery disease, chronic obstructive pulmonary disease, diabetes mellitus, other cardiac disease), scheduled surgical procedure and subspecialty, smoking habits (number of cigarettes per day, years smoking), Fagerstrom score for nicotine dependence. Operative day information to be collected in addition to outcomes listed below: surgical procedure, duration of surgery, anesthesia type (local/monitored anesthesia care/regional/general/combined)

| **Outcome** | **Data Description** | **Likely Distribution** | **Hypothesis Test** |
| --- | --- | --- | --- |
| Smoking cessation (primary outcome) | Proportion | n/a | Fisher’s exact test |
| Smoking reduction | Proportion | n/a | Fisher’s exact test |
| Smoking amount | Cigarettes/day, Continuous  Median (IQR) | Normal or skewed (plot histogram) | Normal: two-sided t-test  Skewed:Wilcoxon rank-sum |
| Unanticipated hospital admission | Proportion | n/a | Fisher’s Exact Test |
| Hospital length of stay | Days  Continuous  Median (IQR) | Skewed | Time-to-event using Cox proportional hazards model; graph Kaplan-Meier plot |
| 8-weeks after randomization: Agreement to (0-7 likert) "I was satisfied with product I was given (e-cigarette or patch) to help me quit" (and similar questions regarding how helpful they found it, and whether they would recommend e-cig/patch) | Likert 0-7, Ordinal, Median (IQR) | Skewed | Wilcoxon rank-sum |
| 8-weeks after randomization: During the 6-weeks after you entered the study, how often did you use the product you were given (e-cigarettes or patch)?  Everyday, most days, a few times a week, once a week, less than once a week, did not use at all | Proportion that used everyday Proportion that did not use at all. | n/a | Fisher’s exact test |
| Change in FEV1, FVC, salivary cotinine | Liters, Continuous (Mean, SD) | Normal | Paired t-test |
| Composite of 30-day postoperative complications and mortality | Proportion | n/a | Fisher’s Exact Test |
| Duration in PACU | Hours  Continuous  Median (IQR) | Skewed | Time-to-event using Cox proportional hazards model; graph Kaplan-Meier plot |

## Qualitative Analysis:

Individual interviews will be performed 8-weeks after randomization to determine patient attitudes towards e-cigarettes and nicotine patches perioperatively. Specifically, we will solicit information from patients regarding their perceptions of how the intervention (e-cigarette or nicotine patch) affected their willingness and/or success at quitting. Thematic and content analysis will be used to identify core concepts and to develop categories for coding interview data. Content analysis will be used to systematically sort and code information based on themes.

## Timetable:

In 2013, the SFVAMC had 3914 cases, averaging 325 cases per month. Roughly 70% of the cases are elective, or around 230 per month. Around 20% are smokers, so each month, we expect to encounter 45 smokers. Since only half are expected to present to preadmission at least 1-week prior to surgery and we anticipate a recruitment rate of 50% of smokers, or around 10 per month. Therefore, we expect pilot study recruitment to take 3 months. The primary end-point data is anticipated to be collected by around 1 month after recruitment finishes, with secondary end-points collected after another month.

## How this pilot study will lead to future funding:

I currently have 70% protected research time as part of the Clinical Instructorship with Research Training offered by the Department of Anesthesia and Perioperative Care at UCSF. I have expertise in the development of perioperative smoking cessation programs in both a clinical and a clinical trials setting. I am currently enrolled in the UCSF Advanced Training in Clinical Research (ATCR) program, with anticipation of completing the Master's degree in clinical research in 2015. The experience and preliminary data gained from this study will be beneficial in terms of both attainment of career development and project-specific research funding. Our intention is to apply to the Tobacco-Related Disease Research Program (Research Project - $375,000) and to the National Institutes of Health (NIH - R21 Exploratory/Developmental Research Grant Program - $275,000) for funding of a full-scale trial. The full-scale trial will be more geared towards Aims 2-4 of this proposal and will again include a head-to-head comparison of e-cigarettes versus nicotine replacement therapy.

## Mentoring plan:

**Research Team:** To conduct the proposed research, we have assembled a highly qualified, multi-disciplinary team of investigators. Together, this team of investigators has the relevance and diversity of expertise to achieve the specific aims of the proposed research plan, and will meet biweekly during recruitment and until study completion.

**Arthur Wallace, MD, PhD**, the primary mentor, is a Professor of Anesthesia and Perioperative Care at UCSF and SFVAMC with expertise in cardiac anesthesia. He is the Director of Perioperative Care Clinic at SFVAMC. Dr. Wallace was one of the developers of perioperative beta blockade and has assisted more than 150 hospitals set up beta blocker programs. He has extensive expertise in implementation of clinical and research protocols in an inpatient setting.

**Mehrdad Arjomandi, MD**, the co-mentor, is an Assistant Professor In-residence of Pulmonary and Critical care Medicine with a joint appointment at UCSF and SFVAMC. He is a graduate of NIH-funded UCSF Clinical Translational Research Institute (CTSI) Advanced Training in Clinical Research (ATCR). He is also a member of UCSF Center for Tobacco Control Research and the Associate Director of the UCSF Human Exposure Laboratory. Drs. Lee, Wallace, and Arjomandi will be responsible for recruitment, enrollment, and management of patients participating in the study.

## Strengths of this research plan:

Perioperative smoking is a widely known clinical problem. This study will address the effectiveness of perioperative interventions to reduce smoking. In addition, it will help contribute to a desperately needed area of smoking cessation and tobacco research by investigating the utility of e-cigarettes as aids to smoking cessation. The research team assembled has the expertise to explore these important areas and complete the aims laid out in this plan in a timely fashion.

# Literature cited:

1. Warner DO. Perioperative abstinence from cigarettes: physiologic and clinical consequences. Anesthesiology 2006;104:356-67.

2. Musallam KM, Rosendaal FR, Zaatari G, et al. Smoking and the risk of mortality and vascular and respiratory events in patients undergoing major surgery. JAMA Surg 2013;148:755-62.

3. Shi Y, Warner DO. Surgery as a teachable moment for smoking cessation. Anesthesiology 2010;112:102-7.

4. Lee SM, Landry J, Jones P, M., Buhrmann O, Morley-Forster P. The effectiveness of a perioperative smoking cessation program: a randomized clinical trial. Anesth Analg 2013;117:605-13.

5. Moller AM, Villebro N, Pedersen T, Tonnesen H. Effect of preoperative smoking intervention on postoperative complications: a randomised clinical trial. Lancet 2002;359:114-7.

6. Lindstrom D, Azodi O, Wladis A, et al. Effects of perioperative smoking cessation intervention on postoperative complications, a randomized trial. . Annals of Surgery 2008;248.

7. Wong J, Abrishami A, Yang Y, et al. A perioperative smoking cessation intervention with varenicline: a double-blind, randomized, placebo-controlled trial. Anesthesiology 2012;117:755-64.

8. Sadr Azodi O, Lindstrom D, Adami J, et al. The efficacy of a smoking cessation programme in patients undergoing elective surgery: a randomised clinical trial. Anaesthesia 2009;64:259-65.

9. Villebro NM, Pedersen T, Moller AM, Tonnesen H. Long-term effects of a preoperative smoking cessation programme. Clin Respir J 2008;2:175-82.

10. Bullen C, Howe C, Laugesen M, et al. Electronic cigarettes for smoking cessation: a randomised controlled trial. Lancet 2013;382:1629-37.

11. Barbeau AM, Burda J, Siegel M. Perceived efficacy of e-cigarettes versus nicotine replacement therapy among successful e-cigarette users: a qualitative approach. Addict Sci Clin Pract 2013;8:5-.

12. Warner DO. Helping surgical patients quit smoking: why, when, and how. Anesth Analg 2005;101:481-7.

13. Sorensen LT. Wound healing and infection in surgery. The clinical impact of smoking and smoking cessation: a systematic review and meta-analysis. Arch Surg 2012;147:373-83.

14. Sorensen LT, Karlsmark T, Gottrup F. Abstinence from smoking reduces incisional wound infection: a randomized controlled trial. Ann Surg 2003;238:1-5.

15. Myles PS, Iacono GA, Hunt JO, et al. Risk of respiratory complications and wound infection in patients undergoing ambulatory surgery: smokers versus nonsmokers. Anesthesiology 2002;97:842-7.

16. Brown DW. Smoking prevalence among US veterans. J Gen Intern Med 2010;25:147-9.

17. Gierisch JM, Straits-Tröster K, Calhoun PS, Beckham JC, Acheson S, Hamlett-Berry K. Tobacco use among Iraq- and Afghanistan-era veterans: a qualitative study of barriers, facilitators, and treatment preferences. Prev Chronic Dis 2012;9:E58-E.

18. Duffy SA, Kilbourne AM, Austin KL, et al. Risk of smoking and receipt of cessation services among veterans with mental disorders. Psychiatr Serv 2012;63:325-32.

19. Singh JA, Hawn M, Campagna EJ, Henderson WG, Richman J, Houston TK. Mediation of smoking-associated postoperative mortality by perioperative complications in veterans undergoing elective surgery: data from Veterans Affairs Surgical Quality Improvement Program (VASQIP)--a cohort study. BMJ Open 2013;3.

20. Thomsen T, Villebro N, Møller AM. Interventions for preoperative smoking cessation. Cochrane Database Syst Rev 2014;3:CD002294-CD.

21. Kai T, Maki T, Takahashi S, Warner DO. Perioperative tobacco use interventions in Japan: a survey of thoracic surgeons and anaesthesiologists. Br J Anaesth 2008;100:404-10.

22. Warner DO, Sarr MG, Offord KP, Dale LC. Anesthesiologists, general surgeons, and tobacco interventions in the perioperative period. Anesth Analg 2004;99:1766-73.

23. Palazzolo D. Electronic cigarettes and vaping: a new challenge in clinical medicine and public heatlh. A literature review. Frontiers in Public Health 2013;1:56(1):1-19.

24. Grana R, Benowitz N, Glantz SA. E-cigarettes: a scientific review. Circulation 2014;129:1972-86.

25. Kadimpati S, Nolan M, Warner DO. Attitudes, beliefs, and practices regarding electronic nicotine delivery systems in patients scheduled for elective surgery. Mayo Clin Proc 2015;90:71-6.

26. Schane RE, Ling PM, Glantz SA. Health effects of light and intermittent smoking: a review. Circulation 2010;121:1518-22.

27. Wallace A. Do deficiencies of endothelial derived relaxing factor contribute to myocardial stunning? J Card Surg 1993;8:325-8.

28. Wallace A, Bellows W, Moores W, et al. Left ventricular pressure-dimension relationships derived from invasive and non-invasive methods in man. Anesth Anal 1994;78:s462.

29. Wallace A, Fontes M, Mathew J, et al. The Association of the Pattern of Use of Anti-Ischemic Agents on Morbidity and Mortality After Coronary Artery Bypass Surgery. ASA Abstracts 2003.

30. Wallace A, Kalra R, Neville S, et al. Audiovisual Distraction in Patients Undergoing Surgery with Regional Anesthesia:. ASA Abstracts 2004.

31. Wallace A, Lam HW, Mangano DT. Linearity, load dependence, hysteresis, and clinical associations of systolic and diastolic indices of left ventricular function in man. Multicenter Study of Perioperative Ischemia (McSPI) Research Group. J Card Surg 1995;10:460-7.

32. Wallace A, Lam HW, Nose PS, Bellows W, Mangano DT. Changes in systolic and diastolic ventricular function with cold cardioplegic arrest in man. The Multicenter Study of Perioperative Ischemia (McSPI) Research Group. J Card Surg 1994;9:497-502.

33. Wallace A, Layug B, Tateo I, et al. Prophylactic atenolol reduces postoperative myocardial ischemia. McSPI Research Group Anesthesiology 1998;88:7-17.

34. Wallace AW, Galindez D, Salahieh A, et al. Effect of Clonidine on Cardiovascular Morbidity and Mortality after Noncardiac Surgery. Anesthesiology 2004;101:284-93.

35. Wallace AW, Ratcliffe MB, Galindez D, Kong JS. L-arginine infusion dilates coronary vasculature in patients undergoing coronary bypass surgery. Anesthesiology 1999;90:1577-86.

36. Wallace AW, Ratcliffe MB, Nose PS, et al. Effect of induction and reperfusion with warm substrate-enriched cardioplegia on ventricular function [In Process Citation]. Ann Thorac Surg 2000;70:1301-7.

37. Hulley S, Cummings S, Browner W, Grady D, Newman T. Appendix 6. In: Designing clinical research: an epidemiologic approach 4th ed. Philadelphia, PA: Lippincott Williams & Wilkins; 2013:73.

38. Konstantinos EF, Riccardo P. Safety evaluation and risk assessment of electronic cigarettes as tobacco cigarette substitutes: a systematic review. Ther Adv Drug Saf 2014;5:67-86.

39. Nides MA, Leischow SJ, Bhatter M, Simmons M. Nicotine blood levels and short-term smoking reduction with an electronic nicotine delivery system. Am J Health Behav 2014;38:265-74.

40. Grana RA, Ling PM. "Smoking revolution": a content analysis of electronic cigarette retail websites. Am J Prev Med 2014;46:395-403.

41. Nitzkin JL. The case in favor of e-cigarettes for tobacco harm reduction. Int J Environ Res Public Health 2014;11:6459-71.

# 16. Budget

**Awarded funding: $18,569 from internal UCSF Department of Anesthesia funds**

**Amount from UCSF RAP: $29,969**

**Total research budget: $48,538**

**Personnel-**Research Assistants

One full-time research assistant (healthcare professional with previous experience in patient interaction) at 40 hours per week and one part-time research assistant at 17.5 hours per week, each at a rate of $25.29/hr will be hired. Two research assistants are needed in order to ensure that the research assistant performing the randomization is different than the one collecting post-randomization data (i.e. to maintain blinding). We anticipate that during the first and last months of the study, we will require fewer hours since few patients will be booked for surgery within the first few weeks and no new patients will be recruited in the final 1-2 months of the study, while outcome data will continue to be collected.

Therefore, the two assistants will overlap by 4.5 months, so there will be 6 months of total coverage.

|  | Hours per week | Hourly rate | Benefits | Weekly rate | Number of weeks | Total cost |
| --- | --- | --- | --- | --- | --- | --- |
| Full-time | 40 | 25.29 | 41.72% | 1433.64 | **20** | $30,106.43 |
|  |  |  |  |  |  | $30,106.43 |

Fringe benefits are calculated using the UCSF established rates of 41.72% for 'Staff Personnel – career'.

Apart from the research assistants, no other salary support is being requested. I (Dr. Lee) have 70% protected research time, as part of the Clinical Instructorship with Research Training, that I will devote in part to this trial.

**Consulting Costs**

UCSF Clinical and Translational Sciences Institute (CTSI) Consultation Services

The Department of Anesthesia at SFVAMC has an in-house biostatistician, Dr. Stephen Takemoto, who can consult regarding statistical issues at no charge. The CTSI provides one hour of free consultation and a further one hour at $140/hour is budgeted for additional study planning and data analysis problems.

**Equipment & Supplies**

Bedfont Scientific piCO+ Smokerlyzer and MIR Spirobank Spirometer

The Smokerlyzer ($620) is used to detect exhaled carbon monoxide, which at levels <10ppm confirms abstinence for the previous 24 hours. The initial purchase includes 12 oval D-pieces that require changing once per month, 25 mouthpieces that require changing after each use, and 25 non-alcohol based disinfection wipes (alcohol-based wipes will damage the sensor). This study will also require 1 box (250) mouthpieces ($37) and 75 additional non-alcohol based disinfecting wipes ($45). The MIR Spirobank Spirometer ($525) and additional mouthpieces ($69) will be used to assess the secondary outcome of change in FEV1 and FVC.

**Other Costs**

**NJOY Electronic nicotine delivery device (e-cigarette)**

The average cost for a 6-week supply of NJOY disposable e-cigarettes for a 1PPD smoker would be 15 e-cigarettes per week, or 3 5-packs per week at $12/5-pack = $36/week or $216 per 6-weeks. An additional $444 is budgeted for those smokers of >1 pack per day who may require additional cartridges.

For all 20 participants, this would cost: $4764

**NRT costs**

When purchased through the VA pharmacy, nicotine replacement therapy patches cost:

$11.13 for 2-week supplies of 7mg and 14mg patches

$17.04 for a 2-week supply of 21mg patches

For smokers >10cig/day, the cost for 6-weeks would be: $17.04 + $11.13 + $11.13 = $39.3/6-weeks

For all 10 participants, this would cost: $393

**Salivary cotinine analysis costs**

Awaiting formal quote from Neal Benowtiz' laboratory. 3 samples per subject = 90 samples total. Budget $2970 assuming $33 per sample.

**Participant incentive**

To encourage adherence to the protocol and follow-up, participants will be given $40 upon completing the study. The total cost of incentives for the 30 patients is $1200.

**Data Management Costs**

The trial will require a computer on which to enter study data, statistical software and database software. Wherever possible, we will use existing hospital, university, or personal resources. Data will be collected and managed on a Microsoft Access database, which is provided behind the VA firewall at no cost to investigators. All data will be stored securely behind the VA firewall, which is encrypted and backed-up regularly.

We anticipate no travel, inpatient, outpatient, or renovation costs to be associated with this study.
